# Stoichiometric traits of stickleback: Effects of genetic background, rearing environment, and ontogeny

**DOI:** 10.1002/ece3.2802

**Published:** 2017-03-18

**Authors:** Miguel Costa Leal, Rebecca J. Best, Dan Durston, Rana W. El‐Sabaawi, Blake Matthews

**Affiliations:** ^1^ Department of Fish Ecology and EvolutionEawag: Swiss Federal Institute of Aquatic Science and TechnologyCentre for Ecology, Evolution and BiogeochemistryKastanienbaumSwitzerland; ^2^ Department of Aquatic EcologyEawag: Swiss Federal Institute of Aquatic Science and TechnologyCentre for Ecology, Evolution and BiogeochemistryKastanienbaumSwitzerland; ^3^ Department of BiologyUniversity of VictoriaVictoriaBCCanada

**Keywords:** allocation, condition, ecological stoichiometry, fish, phosphorus

## Abstract

Phenotypes can both evolve in response to, and affect, ecosystem change, but few examples of diverging ecosystem‐effect traits have been investigated. Bony armor traits of fish are good candidates for this because they evolve rapidly in some freshwater fish populations, and bone is phosphorus rich and likely to affect nutrient recycling in aquatic ecosystems. Here, we explore how ontogeny, rearing environment, and bone allocation among body parts affect the stoichiometric phenotype (i.e., stoichiometric composition of bodies and excretion) of threespine stickleback. We use two populations from distinct freshwater lineages with contrasting lateral plating phenotypes (full vs. low plating) and their hybrids, which are mostly fully plated. We found that ontogeny, rearing environment, and body condition were the most important predictors of organismal stoichiometry. Although elemental composition was similar between both populations and their hybrids, we found significant divergence in phosphorus allocation among body parts and in phosphorus excretion rates. Overall, body armor differences did not explain variation in whole body phosphorus, phosphorus allocation, or phosphorus excretion. Evolutionary divergence between these lineages in both allocation and excretion is likely to have important direct consequences for ecosystems, but may be mediated by evolution of multiple morphological or physiological traits beyond plating phenotype.

## 1 Introduction

There is growing evidence that within‐species phenotypic variation can affect community and ecosystem dynamics in general (Harmon et al., 2009; Lunndsgaard‐Hansen, Matthews, & Seehausen, 2014; Matthews, Aebischer, Sullam, Lundsgaard‐Hansen, & Seehausen, 2016) and biogeochemical cycling of nutrients in particular (Bassar et al., 2010; El‐Sabaawi et al., 2015; Rudman et al., 2015). In aquatic ecosystems, separate bodies of research have documented the effects of fish on ecosystem function and the existence of important phenotypic variation between fish species, but much less work has been done to assess within‐species phenotypic variation and its consequences for ecosystem functioning. For example, fish can affect nutrient dynamics directly by excretion and habitat modification (Knoll, McIntyre, Vanni, & Flecker, 2009; McIntyre et al., 2008) and indirectly by altering the composition and biomass of lower trophic levels (Vanni, Boros, & McIntyre, 2013; Vanni, Layne, & Arnott, 1997). The strength of such consumer‐mediated effects on nutrient cycling likely depends on traits that can vary both among and within species (El‐Sabaawi et al., 2014; Elser & Urabe, 1999), but it remains unclear which traits govern these effects, how variable these traits are among populations, and how they might evolve (Leal, Seehausen, & Matthews, 2017).

Stoichiometric traits, such as the elemental composition of organisms, elemental allocation into specific body parts, or waste production through excretion, are good candidates for studying the effects of organisms on nutrient cycling (Jeyasingh, Cothran, & Tobler, 2014; Leal et al., 2017). Expressing organisms in terms of their elemental phenotype (EP) is useful for identifying “ecosystem effect” traits (Matthews et al., 2011) and for understanding how rapid trait evolution might affect contemporary ecosystem dynamics (Yamamichi, Meunier, Peace, Prater, & Rúa, 2015). Ecological stoichiometry posits that animals maintain relatively constant elemental composition, i.e., are homeostatic (Sterner & Elser, 2002), but there is growing evidence for significant intraspecific, intrapopulation, and intraindividual (i.e., ontogenetic) variability in stoichiometric traits. Intraspecific variability can originate from a combination of both genetic and environmental effects (El‐Sabaawi, Kohler, et al., 2012; Lind & Jeyasingh, 2015). Within individuals, stoichiometric traits can vary based on the balance between nutrient demand and availability throughout ontogeny (Boros, Saly, & Vanni, 2015). Understanding more about the amount, genetic basis, and plasticity of stoichiometric variability of animals can help us understand how phenotypic evolution might affect ecosystem dynamics (Leal et al., 2017; Matthews et al., 2011).

Fish are an excellent system for studying both the determinants and ecosystem consequences of stoichiometric variation (McIntyre et al., 2008; Vanni, Flecker, Hood, & Headworth, 2002). Fish play an important role in phosphorus (P) dynamics in freshwater ecosystems, partly because their bones represent an important P pool (Hendrixson, Sterner, & Kay, 2007). Fish body P demand can affect nutrient recycling via excretion, as predicted by stoichiometric theory (Elser & Urabe, 1999). P‐rich bony structures are also linked to well‐known examples of adaptive traits, such as spines and armor plates (Berner, Moser, Roesti, Buescher, & Salzburger, 2014). Threespine stickleback (*Gasterosteus aculeatus*) populations, for example, vary in their body armor, which can evolve rapidly and has repeatedly been lost when marine populations colonize freshwater environments (Bell, Aguirre, & Buck, 2004). Differences in armor phenotype have been associated with variation in %P and in the ratio between nitrogen and phosphorus (N:P), with fully armored fish showing higher %P and lower N:P than low armored ones (El‐Sabaawi, Warbanski, Rudman, Hovel, & Matthews, 2016).

Here, we use two independent evolutionary lineages of European threespine stickleback from the Baltic (Constance) and French Rhone (Geneva) drainages varying in several traits. For instance, the Baltic lineage shows the fully plated phenotype, small head length, and higher vertebrae number, whereas the French Rhone lineage shows a low‐plated phenotype, large head length, and lower vertebrae number (Berner et al., 2014; Lucek, Roy, Bezault, Sivasundar, & Seehausen, 2010; Roy, Lucek, Bühler, & Seehausen, 2010). These different evolutionary lineages were used in this study to explore how lineage effects compare to environmental and ontogenetic effects on stoichiometric traits. We test whether these two lineages differ in their body elemental content, P allocation to different body parts, and P excretion. If body armor makes up a substantial proportion of total body P, then we expect fully plated fish to be higher in %P while excreting less P (Vanni et al., 2002). Using hybrids with dominant expression of the fully plated phenotype further allows us to test whether stoichiometric differences in body composition, allocation, or excretion are consistent with shared plating phenotype regardless of other genetic and phenotypic differences between fully plated populations (i.e., Constance and hybrids). We also hypothesize that %P increases and N:P decreases with fish length across ontogenetic stages (i.e., juveniles and adults) as P‐rich bony structures make up a larger proportion of fish biomass (Casadevall, Casinos, Viladiu, & Ontanon, 1990) and that body stoichiometry changes with fish condition, which is affected by environmental conditions such as food availability (Sterner & Elser, 2002).

## 2 Materials and Methods

### 2.1 Study species

We used threespine stickleback from two independent lineages in Switzerland, both of which have a recent history of introduction and range expansion into Swiss lakes and streams (Lucek et al., 2010). Lake Constance and Lake Geneva populations (hereafter Constance and Geneva) represent distinct lineages originating from different drainages (Baltic and French Rhone, respectively) and, among other phenotypic differences, display distinct armor phenotypes (Berner et al., 2014; Lucek et al., 2010); Constance fish are fully plated and Geneva fish are low plated. Also note that these two lineages are hybridizing throughout Swiss watersheds, which has been promoting the colonization of new water bodies (Lucek et al., 2010). To compare with these two populations, we also bred and reared F1 hybrids in the laboratory, most of which are fully plated. Although stickleback armor has several components (lateral plates, pelvic girdle, and pelvic spines), we quantified lateral plates to identify the armor phenotype because they have a known genetic basis and pattern of inheritance and there are clear differences between our two ancestral lineages (Lucek et al., 2010). We counted the structural and posterior plates (Bergstrom & Reimchen, 2003) because accurately counting anterior and caudal plates requires staining, which is incompatible with elemental analysis.

### 2.2 Fish breeding, raising environments, and measured traits

**Figure 1:**
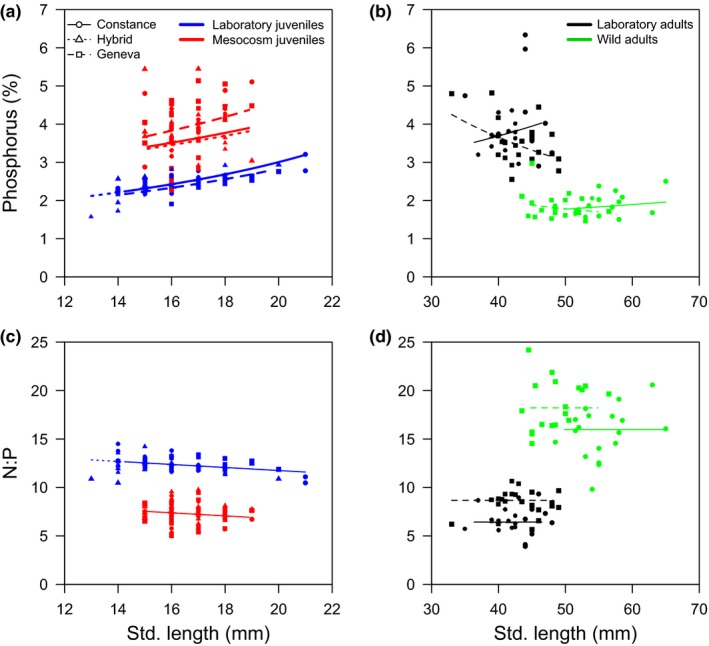
Relationship between elemental phenotype (a, b – %P, c, d – N:P) and fish standard length of juvenile (a, c) and adult fish (b, d) fish. The fitted lines represent the linear model to indicate the slope and intercept of the relationships across all fish types and environmental contexts. Note that no hybrid wild adults are present. Details of the LMs are shown in Table 1

For the goals of this study, we needed fish (1) with different genetic backgrounds and contrasting armor phenotypes, (2) at different ontogenetic stages (i.e., juveniles and adults), and (3) from different environmental contexts that could affect fish condition. To accomplish this, we collected wild adults (group 1) and bred them to create juveniles that were raised for either 2 months (group 2) or 12 months (group 3) in the laboratory. To test the short‐term environmental effects of rearing environment on stoichiometric traits, we also transferred individuals from group 2 into outdoor mesocosms stocked with natural prey communities for 6 weeks (group 4). Note that despite age differences between groups 2 and 4, all juvenile fish showed similar length (Figure 1).

Reproductively mature Constance and Geneva fish were caught at Marina Rheinhof (Altenrhein, Switzerland) and at Les Grangettes (Noville, Switzerland), respectively, in April/May 2014. We used in vitro fertilization with random pairs of a single male and female to produce pure lineage juveniles (30 families of each) and F1 hybrid juveniles (30 families with half using a Constance female and half using a Geneva female in order to generate a F1 population with mixed genetic background). Parents were sacrificed using MS‐222, and nine males and nine females of each lineage, i.e., Constance and Geneva (group 1, hereafter wild adults) were stored at −20°C. Within all other groups, we use stickleback “type” to refer to the three origins (Constance, Geneva, or hybrid). Fertilized clutches of the same type were suspended in well‐aerated 250‐L tanks in groups of 6 and dead eggs were removed daily. After hatching, juveniles were fed with *Artemia* sp. nauplii and zooplankton collected from Lake Lucerne (Switzerland). After 2 months, a total of 60 juveniles (20 of each type; group 2, hereafter referred to as laboratory juveniles) were sacrificed and stored at −20°C. Remaining juveniles were either raised to adulthood (1 year old but not yet reproductively ripe) in the laboratory on a diet of frozen chironomids (group 3, hereafter laboratory adults), or transferred to outdoor mesocosms (1,000 L each) filled with gravel, sand, and water from Lake Lucerne, and inoculated with sediment and zooplankton from Lakes Lucerne, Constance, and Geneva to maximize diversity of pelagic and benthic organisms. After 6 weeks in the mesocosms, a total of 72 juveniles (2–4 fish of each type from each of eight mesocosms, for a total of 24 per type) were sampled and stored at −20°C (group 4, hereafter referred to as mesocosm juveniles). All fish were handled in accordance with permits issued by the Lucerne cantonal authority (Switzerland).

Fish from all groups were processed in the laboratory to characterize standard length, plating phenotype (for adult fish only), dry weight, and whole body elemental content and stoichiometry (%P and N:P). Body condition of all fish was calculated by regressing the log‐transformed dry weight of each fish against the log‐transformed standard length and taking the residuals. We did this separately for juveniles and adults, but within each life stage found that all types had the same slope for the length vs. weight relationship. The residuals were used as a proxy for body condition (weight of fatty and muscular tissue for a skeleton of a constant length). The validity of using residuals to indicate condition was supported by comparing the residuals with the hepatosomatic index (Bolger & Connolly, 1989) for a subset of 30 adult fish. This index is defined as the ratio of liver weight to body weight and provides an indication of energy reserves in an animal and was correlated with the length–weight residuals (Pearson correlation: *t *=* *4.1, *df *=* *18, *p *<* *.01). In order to analyze fish body elemental content (%P) and stoichiometry (N:P), all internal organs were removed, and then, fish were freeze‐dried, weighed, and ground using a tissue lyser (TissueLyser II, Qiagen, Hombrechtikon, Switzerland) with tungsten beads (Qiagen, Hombrechtikon, Switzerland). The only exception was fish from group 3 (laboratory adults) which were first used to measure P excretion.

### 2.3 Phosphorus excretion

Laboratory adults of each type (*n *=* *20) were starved over 24 hrs and acclimatized to tap water (21°C) in separate containers for 2 hrs to minimize manipulation stress and any husbandry tank‐related bias. Fish were then incubated in 0.5‐L aquaria for 60 min. Water samples (10 ml) for total dissolved P were collected before and after incubation and measured using the molybdate method (Parsons, Maita, & Lalli, 1984). After the incubation trials, fish were sacrificed using MS‐222 as previously described and stored at −20°C and processed as described in the previous section (2.2) for measuring whole body %P and N:P.

### 2.4 Elemental allocation among body parts

A different set of 10 laboratory‐reared adults from of each type (Constance, Geneva, and hybrids) were dissected into multiple parts for measuring elemental allocation: head, gill arch, liver, gut, gonads, pelvic girdle, muscle, skin (including plates), and other bone structures and fins. All fish and body part samples were then freeze‐dried over 48 hr, weighed, and ground as previously described (see section 2.2).

### 2.5 Analytical protocols for measuring %P and N:P

Five to ten milligrams of ground tissue was weighed, diluted in 20 ml potassium peroxodisulfate (10 g K_2_O_8_S_2_, 1.5 g NaOH, 1L Milli‐Q water), and autoclaved for 2 hr at 121°C for P digestion. P and N concentrations were determined colorimetrically using a continuous flow analyzer (Skalar Analytical B.V., Breda, the Netherlands) following the ammonium molybdate method (Parsons et al., 1984) and standard procedure ISO 13395:1996, respectively. Drift and baseline corrections were programmed each 18 samples. P recoverability of 95% was determined with bone meal reference material (NIST 1486). N and P concentrations was used to calculate molar N:P.

### 2.6 Statistical analysis

We assessed factors contributing to individual elemental and stoichiometric variability (%P and N:P) using general linear models (LMs) for each ontogenetic stage (juveniles and adults). Specifically, we used these LMs to test, for each ontogenetic stage, how either fish length or condition interacted with rearing environment (laboratory, mesocosms, wild) and fish type (Constance, Geneva, hybrid) to predict %P or N:P. We conducted this analysis separately for each ontogenetic stage. Additionally, we explicitly tested for effects of environment and fish type on body condition, which we predicted could mediate effects on EP. Although sex could also have an effect on stoichiometric traits, we did not distinguish between males and females because sex was a weak predictor of body stoichiometry in adult fish ((*n*_males_ **=* *48, *n*_females_ **=* *53): *F *=* *0.8, *p *=* *.37). Residuals for all models were tested for normality and heterogeneity of variance, and %P was inverse transformed to meet these assumptions.

We compared P content of each body part among fish types with one‐way ANOVAs. Tukey's HSD post hoc test was used when statistical differences were observed (*p *<* *.05). A linear regression was used to test how fish type and P excretion predicted organismal stoichiometric traits (i.e., %P and N:P, response variables), and residuals tested for normality and heterogeneity of variance. We also calculated a correlation matrix based on the Pearson correlation among P content of each body part to assess the P allocation relationship among different body parts. All statistical analyses were performed using R (R Core Team 2015).

## 3 Results

### 3.1 Environmental and ontogenetic variation

Overall, environment significantly affected the EP for both juveniles and adults, whereas fish‐type effects were often length and/or environment dependent (Table 1). %P was higher and N:P lower in laboratory adults compared to wild adults, and in juveniles, this was reversed, with laboratory juveniles having lower %P and higher N:P than mesocosm juveniles (Figure 1). Within the juveniles, %P increased with length, with slightly different slopes for different environment × type combinations, and N:P decreased with length (Figure 1a,b, Table 1). Within the adults, the two types showed somewhat opposite effects of length on %P (Figure 1a) and were consistently different in N:P, which was higher in Geneva regardless of environment or length (Figure 1d).

**Table 1:** Ontogeny‐specific general linear model (LM) analysis for stickleback elemental composition (%P) and N:P stoichiometry with fish length as covariate and fish type (Constance, Geneva, hybrid) and environment (laboratory vs. mesocosms or wild) as main effects together with two‐way and three‐way interactions. Note that adult hybrids were not considered for the adult LM, as they were only available for the laboratory environment. A total of 60 laboratory juveniles (20 per type), 72 mesocosms juveniles (24 per type), 66 laboratory adults (22 per type), and 36 wild adults (18 per type) were analyzed. Bold values denote significant differences

Ontogeny	Predictor	ndf,ddf	Response: %P		Response: N:P	
			*F*	*p*	*F*	*p*
Juveniles	Length	1,131	28.04	**.00**	10.51	**.00**
	Type	2,131	0.48	.62	0.39	.68
	Environment	1,131	223.22	**.00**	878.85	**.00**
	Length × type	2,131	0.21	.81	1.55	.22
	Length × environment	1,131	1.73	.19	0.27	.87
	Type × environment	2,131	2.43	.09	2.52	.08
	Length × type × environment	2,131	4.18	**.02**	0.26	.78
Adults	Length	1,101	0.31	.58	2.64	.11
	Type	1,101	1.07	.31	23.30	**.00**
	Environment	1,101	137.58	**.00**	174.66	**.00**
	Length × type	1,101	4.33	**.04**	0.02	.90
	Length × environment	1,101	0.66	.42	2.19	.14
	Type × environment	1,101	4.06	**.05**	2.47	.12
	Length × type × environment	1,101	1.41	.24	0.68	.68

**Figure 2:**
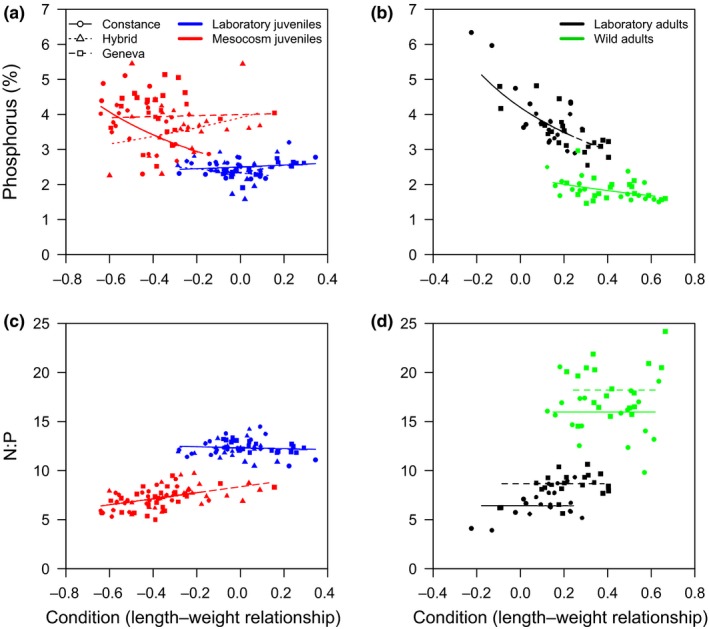
Relationship between elemental phenotype (a, b – %P, c,d – N:P) and fish condition of juvenile (a, c) and adult fish (b, d) fish. The fitted lines represent the linear model to indicate the slope and intercept of the relationships across all fish types and environmental contexts. Note that condition of juvenile and adult fish was estimated using the residuals of different length–weight regressions and that no hybrid wild adults are present. Details of the LMs are shown in the Supporting Information

As the strong effects of environment on EP could be acting via body condition, we assessed the drivers of body condition and found that juvenile condition was higher in the laboratory than the mesocosms (Figure 2a,c) with the magnitude of this contrast depending on fish type (Table S1). The reverse was observed in adults; laboratory adults had lower condition than wild adults regardless of type (Table S1, Figure 2b,d). We also found that overall condition contrasts across environments appeared consistent with contrasts in the EP. For both juveniles and adults, the environment producing high condition fish also produced fish with lower %P and higher N:P (Figure 2). However, environmental effects on %P could not be explained purely via environmental effects on condition because fish types varied in their responses (Table 2). For juveniles, effects of condition on %P depended on fish type and its rearing environment (Figure 2a). However, Constance fish showed both the largest environmental effect on condition and the clearest resulting decrease in %P. In addition, all mesocosm juveniles showed increased N:P with increased condition, but this linear relationship did not extend to the laboratory fish. In the laboratory adults, we found the predicted decrease in %P with increasing fish condition regardless of fish type, but condition had no effect on N:P (Figure 2c,d, Table 2).

**Table 2:** Ontogeny‐specific general linear model (LM) analysis for stickleback elemental composition (%P) and N:P stoichiometry with fish condition as covariate and fish type (Constance, Geneva, hybrid) and environment (juvenile and mesocosms or wild) as main effects together with two‐way and three‐way interactions. Note that adult hybrids were not considered for the adult LM, as they were only available for the laboratory environment. A total of 60 laboratory juveniles (20 per type), 72 mesocosms juveniles (24 per type), 66 laboratory adults (22 per type), and 36 wild adults (18 per type) were analyzed. Bold values denote significant differences

Ontogeny	Predictor	ddf,ndf	Response: %P		Response: N:P	
			*F*	*p*	*F*	*p*
Juveniles	Condition	131,1	0.08	.77	8.20	**.01**
	Type	131,2	1.72	.18	0.16	.85
	Environment	131,1	92.06	**.00**	306.93	**.00**
	Condition × type	131,2	2.58	**.08**	1.03	.36
	Condition × environment	131,1	1.89	.17	14.25	**.00**
	Type × environment	131,2	3.81	**.03**	1.47	.24
	Condition × type × environment	131,2	3.82	**.03**	0.60	.55
Adults	Condition	101,1	27.80	**.00**	1.64	.21
	Type	101,1	0.01	.95	19.26	**.00**
	Environment	101,1	159.00	**.00**	240.24	**.00**
	Condition × type	101,1	0.38	.54	0.91	.34
	Condition × environment	101,1	0.18	.67	2.71	.10
	Type × environment	101,1	0.10	.76	0.65	.42
	Condition × type × environment	101,1	0.22	.65	2.48	.12

### 3.2 Phosphorus allocation

**Figure 3:**
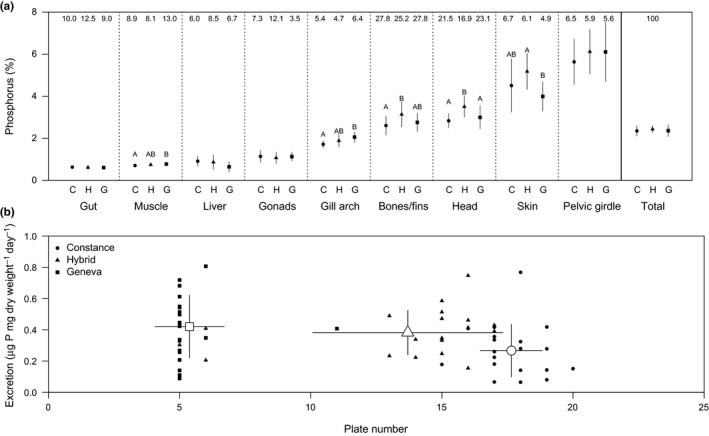
Average (± standard deviation) phosphorus content (%) of each body part and total body (expressed as P content of each body part relative to total body dry weight) for laboratory‐reared fish from Constance (C), Geneva (G), and hybrid (H) types (a). Significant differences among types (*p *<* *.05) are noted with different letters, and the average proportion (%) of each body part (expressed as dry weight of each body part relative to total body dry weight) for each fish type is also shown at the top of each panel. The relationship between phosphorus excretion and plate number is also shown (b), together with average (± standard deviation) plate number and phosphorus excretion for each type

Although %P was similar among fish types (Table 1), significant differences were recorded for particular tissues with contrasting patterns among types for soft and bony tissues, particularly between the parental populations and hybrids (Figure 3a). Bony tissues ranged from 3 to 6%P and were notably higher than soft tissues (0.6 to 2%P). P allocation differences among types varied among tissues; hybrids showed intermediate %P of muscle and gill arch, whereas %P of bones/fins, head, and skin were higher in hybrids than in non‐hybrids. This %P variability among body parts may not reflect total P allocation due to biomass differences among body parts (Table S2). For instance, head and pelvic girdle represent important P pools (25.7 and 15.7% of total body P, respectively). Skin, which includes lateral plates, represents an average of 10.5% of the total body P and allocation of P to skin was lower in low‐plated Geneva than in fully plated Constance or hybrid fish (Table S2). The relationship between the total P content among body parts of all fish revealed that P content variation in all body parts varied concomitantly apart from skin, which did not significantly correlate with any other body part (Table S3).

### 3.3 Phosphorus excretion

P excretion differed among types (LM: *F*_*2,65*_ **=* *5.24, *p *<* *.01) but was not explained by body %P (LM: *F*_*1,65*_ **=* *0.59, *p *=* *.45), which was similar among fish types (LM: *F*_*2,65*_ **=* *2.19, *p *=* *.12). Constance fish (0.25 ± 0.18 μg P mg dry weight^−1^ day^−1^) excreted significantly less P than both Geneva (0.42 ± 0.20 μg P mg dry weight^−1^ day^−1^) and hybrids (0.38 ± 0.14 μg P mg dry weight^−1^ day^−1^) (Tukey's HSD, *p *<* *.05).

## 4 Discussion

Understanding the nature and strength of organismal effects on their environment is critical for understanding reciprocal interactions between phenotypic evolution and ecosystem dynamics. Such interactions depend on the predictability of links between functional phenotypes and environmental effects. Here we show that ontogenetic and environmental effects on organismal stoichiometry are larger than evolved genetic differences between populations and are partly mediated by fundamental aspects of fish condition. In addition, the population effects on stoichiometry were not completely consistent with phenotypic differences in body armor.

For fish raised only under consistent laboratory conditions, large ontogenetic differences were observed and followed the prediction of increasing %P and decreasing N:P with growth from juvenile to adult (Figure 1), which is usually associated with bone development (Elser, Dobberfuhl, MacKay, & Schampel, 1996). The same pattern was also observed within juveniles of increasing length but with type‐ and environment‐specific effects (Figure 1a). Elemental variability within each ontogenetic stage also showed similar magnitudes of environmental effects, which are likely associated with variation in basal resource availability (Dickman, Newell, Gonzalez, & Vanni, 2008; El‐Sabaawi, Zandonà, et al., 2012). Wild adults had access to nutritional variety from zooplankton and benthic invertebrates, whereas laboratory fish were restricted mostly to chironomids. For the juveniles, mesocosm fish had access to a greater variety of foods, but likely lower prey abundance than the laboratory fish. These differences may have also contributed to the condition differences between laboratory and wild or mesocosm fish driven by a differential development of soft and bony tissues. For the adults, reproductive stage may also have affected condition, as the wild adults were reproductively mature and may have had reproduction‐related energy reserves. Although we are uncertain of the underlying cause of variation in condition, which may be associated with high lipid storage in high condition fish (Karimi, Fisher, & Folt, 2010), condition explained some but not all of the EP variability. Despite broad consistency in environmental contrasts in condition and EP (lower %P and higher N:P in environments with high condition fish, Figure 2), within the juveniles variability in condition among fish types superseded consistent linear effects of body condition on %P and N:P across environments (Figure 2).

We expected that fully plated fish would show higher %P based on previous studies (El‐Sabaawi et al., 2016; Vanni et al., 2002). It is important to note that the study by Vanni et al. (2002) analyzed different freshwater fish and amphibian species, whereas the fully plated stickleback fish studied by El‐Sabaawi et al. (2016) were from marine populations. In our study, contrary to our initial expectation, we observed small and inconsistent EP differences due to plating phenotype. While the P content of body armor in the marine populations assessed by El‐Sabaawi et al. (2016) and the Constance population here analyzed could be different, we found a relatively low P content of lateral plates (~10.5% of total P pool) in comparison with other bony tissues (Table S2) and this might explain the weak link between body armor and %P variation. Indeed, the observed differences in P allocation were often opposite (i.e., negatively related) to the predicted positive relationship between %P and plate phenotype (Figure 2a). Similar %P between fully plated Constance and hybrids would be expected if total body P was directly determined by plating phenotype. However, %P content of most body parts was similar between fully plated Constance and low‐plated Geneva fish (Figure 2a). It has also been hypothesized that full body armor would cause a trade‐off in P allocation, such that when more P is needed for armor traits, then less P is available for other tissues (Jeyasingh et al., 2014). However, the covariation of total P content among all tissues and in the same direction, with the exception of skin (Table S3), suggests no trade‐off in P allocation.

Stoichiometric theory predicts that the EP affects nutrient excretion through a negative relationship between excretion and body stoichiometry (Elser & Urabe, 1999). It has also been suggested that excretion stoichiometry in fish is largely controlled by food and not body composition (Pilati & Vanni, 2007). Our results reject the first hypothesis, as no relationship was observed for P excreted and body %P among fish types. This could suggest that P is not limiting in the diet, but this would require additional study of P uptake and enzyme activity (Sullam et al., 2015). Additionally, results suggest that plating phenotype is not directly associated with P excretion (Figure 3b). Although it was initially expected that fully plated fish would show higher P demand and consequently lower P excretion, this pattern was not observed as fully plated hybrid fish excreted P similarly to low‐plated Geneva fish. This could be explained by an effect of plating phenotype (or other morphological trait associated with plate number) on P acquisition but not P demand and excretion. It is also possible that the genetic effects of the mixed genetic background of hybrids on P excretion may exceed the direct effects from differences in nutrient demand determined by organismal stoichiometry. Population differences in nutrient excretion will result in population‐specific impacts on aquatic ecosystems, particularly in hybrid zones between lineages with different armor phenotypes (Lucek et al., 2010; Roy, Lucek, Walter, & Seehausen, 2015). This is because differential excretion can impact the availability of P, a key limiting nutrient, and therefore, the productivity and composition of producer and herbivore trophic levels (Declerck et al., 2015). Such consequences to ecosystem processes can ultimately feed back to select for nutrient requirement of consumers (Yamamichi et al., 2015).

Variation in stickleback armor has been extensively studied, but the ecological consequences of such evolution are still poorly explored, which is particularly relevant for the interaction between ecological and evolutionary processes and the potential for reciprocal feedback on each other (Hendry, Peichel, Matthews, Boughman, & Nosil, 2013). We show that rearing environment and ontogeny are better predictors of organismal stoichiometry than genetic background. Additionally, plating phenotype showed no effect on organismal stoichiometry, probably because plates make up a small fraction of total P. We also show that excretion and fish stoichiometry are decoupled and are not simply defined by plating phenotype or by its genetic background, thereby suggesting that the traditional approach linking excretion with body elemental content does not elucidate its mechanistic driver. A better understanding of nutrient allocation may provide new insights on the functional effects of body armor evolution, particularly if one addresses P‐rich morphological tissues such as pelvic girdle or head that comprise a large fraction of total body P and may explain the variation of whole body stoichiometry. Additionally, and in order to better understand the link between armor evolution, organismal stoichiometry and nutrient recycling, it is important to fully explore the multidimensionality of traits underscoring the EP, i.e., the composition, acquisition, assimilation, allocation, and excretion of elements (Jeyasingh et al., 2014). Such broader stoichiometric approach will improve our understanding of how phenotypic evolution affects ecosystems, and which phenotypic traits govern such ecosystem effects.

## Conflict of Interest

None declared.
