# Supplementary material for: Stoichiometric traits of stickleback: Effects of genetic background, rearing environment, and ontogeny
Source: Ecol Evol. 2017 Mar 18;7(8):2617–25. doi: 10.1002/ece3.2802 (PMC5395448; doi:10.1002/ece3.2802)
Supplement: Supplementary file 1 [file ECE3-7-2617-s001.docx]

Sup. Table 1. Ontogeny-specific general linear model (LM) analysis to predict stickleback body condition from fish type (Constance, Geneva, hybrid) and environment (juvenile and mesocosms or wild) together with their interaction. A total of 60 lab juveniles (20 per type), 72 mesocosms juveniles (24 per type), 66 lab adults (22 per type) and 36 wild adults (18 per type) were analysed. Significant differences are marked (*).

| Ontogeny | Predictor | Response: Body condition | | |
| --- | --- | --- | --- | --- |
|  |  | ndf,ddf | *F* | *p* |
| Juveniles | Type | 1,131 | 8.45 | 0.00* |
|  | Environment | 1,131 | 210.94 | 0.00* |
|  | Type×Environment | 2,131 | 7.19 | 0.00* |
| Adults | Type | 1,101 | 4.91 | 0.03* |
|  | Environment | 1,101 | 55.23 | 0.00* |
|  | Type×Environment | 1,101 | 0.87 | 0.36 |

Sup. Table 2. Average (± st. deviation) phosphorus allocation to each body part (P content of each body part relative to whole body P) for each fish type. Data calculated from phosphorus content (%) and biomass of each body part. A total of 30 lab adult fish (10 per type) were analysed.

| Body part | Phosphorus allocation (%) | | |
| --- | --- | --- | --- |
|  | Constance | Hybrid | Geneva |
| Gut | 2.44 ± 0.53 | 3.10 ± 0.84 | 2.33 ± 0.43 |
| Muscle | 2.68 ± 0.85 | 2.51 ± 0.70 | 4.31 ± 0.89 |
| Liver | 2.37 ± 0.82 | 2.95 ± 0.93 | 1.79 ± 0.49 |
| Gonads | 3.44 ± 1.66 | 4.53 ± 3.19 | 1.56 ± 1.37 |
| Gill arch | 3.85 ± 1.07 | 3.83 ± 1.81 | 5.86 ± 1.42 |
| Bones/Fins | 32.53 ± 8.35 | 32.18 ± 3.62 | 32.57 ± 6.54 |
| Head | 23.64 ± 3.86 | 24.27 ± 2.10 | 29.05 ± 4.51 |
| Skin | 11.18 ± 6.45 | 11.98 ± 4.29 | 8.24 ± 1.77 |
| Pelvic Girdle | 17.87 ± 5.69 | 14.64 ± 2.27 | 14.55 ± 3.41 |

Sup. Table 3. Correlation matrix for the phosphorus content of fish body parts (significant correlations noted with *).

|  | Pelvic girdle | Head | Skin | Muscle | Liver | Gill arch | Gonads | Gut | Bones/ fins |
| --- | --- | --- | --- | --- | --- | --- | --- | --- | --- |
| Pelvic girdle | - |  |  |  |  |  |  |  |  |
| Head | 0.66* | - |  |  |  |  |  |  |  |
| Skin | 0.15 | 0.01 | - |  |  |  |  |  |  |
| Muscle | 0.48* | 0.56* | -0.04 | - |  |  |  |  |  |
| Liver | 0.64* | 0.57* | 0.18 | 0.17 | - |  |  |  |  |
| Gill arch | 0.40* | 0.61* | -0.11 | 0.61* | 0.05 | - |  |  |  |
| Gonads | 0.50* | 0.49* | 0.05 | 0.02 | 0.93* | -0.10 | - |  |  |
| Gut | 0.59* | 0.34* | 0.20 | 0.21 | 0.59* | 0.15 | 0.44* | - |  |
| Bones/Fins | 0.57* | 0.42* | 0.11 | 0.16 | 0.48* | 0.19 | 0.37* | 0.46* | - |
